# Supplementary material for: Analysis of PPARγ Signaling Activity in Psoriasis
Source: Int J Mol Sci. 2021 Aug 10;22(16):8603. doi: 10.3390/ijms22168603 (PMC8395241; doi:10.3390/ijms22168603)
Supplement: Supplementary file 1 [file ijms-22-08603-s001.zip › Supplemental materials_Analysis of PPARg signaling activity in psoriasis/Pathway models/Models images and html files/Differentiation of psoriatic T cells/7383.html]

MAP3K1


# Protein MAP3K1

|  |  |
| --- | --- |
| URN | urn:agi-llid:4214 |
| Total Entities | 0 |
| Connectivity | 1362 |
| Name | MAP3K1 |
| Description | mitogen-activated protein kinase kinase kinase 1, E3 ubiquitin protein ligase |
| Notes | The protein encoded by this gene is a serine/threonine kinase and is part of some signal transduction cascades, including the ERK and JNK kinase pathways as well as the NF-kappa-B pathway. The encoded protein is activated by autophosphorylation and requires magnesium as a cofactor in phosphorylating other proteins. This protein has E3 ligase activity conferred by a plant homeodomain (PHD) in its N-terminus and phospho-kinase activity conferred by a kinase domain in its C-terminus. [provided by RefSeq, Mar 2012] |
| Primary Cell Localization | Cytoplasm |
| Class | Protein kinase |

---

|  |  |
| --- | --- |
| Pathway | Osteoarthritis |
|  | Skin Aging |
|  | Melatonin Effects on Circadian Cycle |
|  | Dopamine Receptors Signaling |
|  | Serotonin Receptors Signaling |
|  | Thrombopoietin Receptors Signaling in Platelet Maturation |
|  | T-Cell Dependent B-Cell Activation |
|  | T-Cell Maturation (Hypothesis) |
|  | Th17-Cell Differentiation |
|  | Plasmin Effects in Inflammation |
|  | Platelet Activation via Adhesion Molecules |
|  | Scavenger Receptors in Platelet Activation |
|  | Bradykinin Effects in Inflammation |
|  | Toll-like Receptor Independent Sterile Inflammation |
|  | MC1R in anti-Inflammatory Signaling |
|  | Leukotriene Effect on Vascular Endothelial Cell Response |
|  | Leukotriene Effect on Airway Smooth Muscle Response |
|  | Basophil Activation |
|  | Mast-Cell Activation without Degranulation through TNFSF8 Signaling |
|  | Mast-Cell Activation via IgE Signaling |
|  | Mast-Cell Activation without Degranulation |
|  | Mast-Cell Activation without Degranulation through IL33/IL1RL1 Signaling |
|  | Mast-Cells De Novo Synthesized Mediators via IgE Independent Signaling |
|  | Mast-Cell Activation without Degranulation through IL1R1 and TLR Signaling |
|  | Macrophage M1 Lineage |
|  | CD16/CD14 Proinflammatory Monocyte Activation |
|  | anti-Inflammatory Function of Macrophage M2 Lineage |
|  | Neutrophil Recruitment and Priming |
|  | Neutrophil Degranulation via FPR1 Signaling |
|  | Vascular Smooth Muscle Cell/Pericyte Migration and Proliferation |
|  | Vascular Endothelial Cell Activation by Cytokines |
|  | Vascular Endothelial Cell Activation by Growth Factors |
|  | Nociception Expression Targets Signaling |
|  | EDNRA/B -> Vascular Motility |
|  | ADRA1A -> IL6 Production |
|  | ADRA1 -> Prostaglandin Generation |
|  | BDKRB1/2 -> Prostaglandin Generation |
|  | CNR1/2 -> IL1B/2/4/6/10 Production |
|  | DRD2 Expression Targets |
|  | CHRM1 -> IL2 Production |
|  | PTGER2/3 -> Inflammation-Related Expression Targets |
|  | HTR1 -> IL6 Production |
|  | HTR5 -> TNF Production |
|  | HTR1 -> Membrane Transport |
|  | IL1B Expression Targets -> Nociception |
|  | Melatonin in Cell Survival and Antioxidant Response |
|  | Circadian Clock Genes in Suprachiasmatic Nuclei Neurons |
|  | Scavenger Receptor OLR1 in Inflammation Related Endothelial Dysfunction |
|  | Lymphocyte Mediated Myocardial Injury in Myocarditis |
|  | Sebocyte Proliferation in Acne Vulgaris |
|  | Inflammatory Reaction in Acne Vulgaris |
|  | Keratinocyte Proliferation in Acne Vulgaris |
|  | Mast-Cells Activation in Atopic Dermatitis |
|  | IL17 Signaling in Psoriasis |
|  | T-Cells Differentiation Block in Psoriasis |
|  | Th17-Cell Function in Systemic Lupus Erythematosus |
|  | B-Cell Activation in Systemic Lupus Erythematosus |
|  | Vitiligo |
|  | T-Cell Cytotoxic Response against Melanocytes in Vitiligo |
|  | Apoptosis of Melanocyte in Vitiligo |
|  | Humoral Immunity in Vitiligo |
|  | Toll-like Receptors in beta-Cell |
|  | Proteins Involved in Diabetes Mellitus Type 2 |
|  | Tubular Cell Dysfunction in Progressive Diabetic Nephropathy |
|  | Endothelial Cell Dysfunction in Early Diabetic Nephropathy |
|  | Mesangial Cell Dysfunction in Diabetic Nephropathy |
|  | Microangiopathy in Diabetic Neuropathy |
|  | Insulin Resistance in Hepatocytes |
|  | beta-Cell Destruction through Cytokines in Diabetes Mellitus |
|  | GNRH Signaling Impairment in Hypogonadotrophic Hypogonadism |
|  | Hypoparathyroidism, Primary |
|  | CD4+T-Cell Response in Celiac Disease |
|  | OipA Signaling in Helicobacter Pylori Infection |
|  | Antiphospholipid Antibodies in Endothelial Cell |
|  | B-Cell Acute Lymphoblastic Leukemia |
|  | B-Cell Chronic Lymphocytic Leukemia |
|  | Chronic Myeloid Leukemia |
|  | Proteins Involved in Chronic Myeloid Leukemia |
|  | Follicular Lymphoma |
|  | Osteoclast Activation in Multiple Myeloma |
|  | Multiple Myeloma |
|  | Antiphospholipid Antibodies and Endothelial Cell Interaction |
|  | T-Cell Acute Lymphoblastic Leukemia |
|  | IL33 Signaling Related Eosinophilia in Asthma |
|  | Airway Smooth Muscle Cell High Rate Proliferation |
|  | IgE Induces Airway Smooth Muscle Cell Proliferation |
|  | Basophil Activation in Asthma |
|  | Mast-Cell Activation in Asthma |
|  | Th17-Cell Differentiation in Asthma |
|  | Mucin Production in Goblet Airway Epithelial Cells |
|  | CFTR Facilitates Oxidative Stress in Airway Epithelium in CF |
|  | Osteoclast Activation in Gout |
|  | TNF and IL1B Induce Metalloproteinase Synthesis in Osteoarthritis |
|  | IL1B Induced Arthralgia |
|  | Th17-Cell and Th1 Immune Responsein Psoriatic Arthritis |
|  | Synovial Fibroblast Activation in Psoriatic Arthritis |
|  | Keratinocyte Activation in Psoriatic Arthritis |
|  | Osteoclast Activation in Rheumatoid Arthritis |
|  | Synovial Fibroblast Activation by Citokines in Rheumatoid Arthritis |
|  | Autocrine Cytokine/Chemokine Loops in Systemic Scleroderma |
|  | B-Cells Function in Systemic Scleroderma |
|  | Skin Fibrosis |
|  | Dentin Formation Disruption |
|  | CCR5 Signaling in Macrophages |
|  | Th17-Cell Numbers Reduction in HIV |
|  | IL1 Receptor Antagonist (IL1RN) Deficiency (DIRA) |
|  | IL36 Receptor Antagonist (IL1F5) Deficiency (DITRA) |
|  | Osteoclast Activation in Postmenopause |
|  | Synaptic Exocytosis in Dementia |
|  | Glioma Invasion Signaling |
|  | Growth Factor Signaling in Neuroblastoma |
|  | Neuroblastoma |
|  | Microglia and Motor Neuron Interaction Dysregulation |
|  | Proteins Involved in Breast Cancer Related to WNT Signaling Pathway |
|  | WNT Signaling in Breast Cancer |
|  | Angiogenesis in Endometriosis |
|  | Subepithelial Fibroblasts in Middle Ear Cholesteatoma |
|  | HPV E5 Regulates EDNRA and EGFR Signaling Pathway in Keratinocytes |
|  | Prostate Cancer |
|  | VEGFA in Neovascular Glaucoma |
|  | Cytokines Trigger Otitis Media |
|  | MUC5AC Expression by NTHi and S. Pneumoniae |
|  | Alveolar Macrophage Negative Regulation Declined in COPD |
|  | Alveolar Epithelial Cell Dysfunction in COPD |
|  | Mucin Hyperproduction in Goblet and Mucous Cells |
|  | WNT Signaling in Cystic Kidney Disease |
|  | Polycystins Mutation Effects in Cystic Kidney Disease |
|  | Plasmin Role in Glomerulonephritis |
|  | Mesangial Cell Dysfunction in Glomerulonephritis |
|  | Interstitial Fibroblasts in Pyelonephritis |
|  | CD38/CD3 -> JUN/FOS/NF-kB Signaling in T-cell Proliferation |
|  | CD38/CD19 -> JUN/FOS/NF-kB Signaling in B-cell Proliferation |
|  | S100P Expression Target |
|  | HMGB1 Expression Targets |
|  | TTR Expression Targets |
|  | S100A Expression Targets |
|  | S100B Expression Targets |
|  | ANGPT1 -> CREB/CREBBP Expression Targets |
|  | ANGPT2 -> AP-1/CREBBP/MYC Expression Targets |
|  | AREG -> NFATC Expression Target |
|  | AREG -> CREB/CREBBP Expression Targets |
|  | AREG -> NCOR2 Expression Targets |
|  | AREG -> AP-1 Expression Targets |
|  | Acetylcholine Expression Targets |
|  | Dopamine/Gi Expression Targets |
|  | BTC -> NFATC Expression Targets |
|  | BTC -> AP-1/ATF/CREB Expression Targets |
|  | Frizzled Receptors -> ARRB1/ARRB2 non-Canonical Signaling |
|  | CD72 -> NF-kB Expression Targets |
|  | CD8 -> NF-kB Expression Targets |
|  | CD80 -> NFATC Expression Targets |
|  | CD86 -> NFATC Expression Targets |
|  | Complement Component GPCRs Signaling |
|  | Clozapine Induced Granulocytopenia |
|  | EGF -> AP-1/ATF Expression Targets |
|  | EGF -> CREB/CREBBP/ELK/SRF/MYC Expression Targets |
|  | EGF -> MEF/MYOD/NFATC Expression Targets |
|  | EGF -> TP53 Expression Targets |
|  | EGF -> NCOR2 Expression Target |
|  | EGFR -> NCOR2 Signaling |
|  | EGFR/ERBB3 -> MEF/MYOD/NFATC/MYOG Signaling |
|  | EGFR/ERBB2 -> TP53 Signaling |
|  | EGFR -> AP-1/ATF2 Signaling |
|  | EGFR -> AP-1/CREB/ELK/SRF/MYC Signaling |
|  | EREG -> CREB Expression Target |
|  | EREG -> AP-1/ATF Expression Targets |
|  | Erythropoietin -> AP-1/MYC/CREB Expression Targets |
|  | F2 -> AP-1/CREB/ELK/SRF/SP1 Expression Targets |
|  | PLG -> AP-1/CREB/ELK/SRF/SP1 Expression Targets |
|  | FGF9 -> AP-1/CREB/MYC Expression Targets |
|  | FGF10 -> AP-1/CREB/CREBBP/MYC Expression Targets |
|  | FGF1 -> AP-1/CREB/ELK/SRF/MYC Expression Targets |
|  | FGF1 -> NCOR2 Expression Target |
|  | FGF8 -> AP-1/CREB/MYC Expression Targets |
|  | FGF18 -> AP-1/CREB Expression Targets |
|  | FGF23 -> NCOR2 Expression Targets |
|  | FGF2 -> NCOR2 Expression Targets |
|  | FGF4 -> AP-1/MYC Expression Targets |
|  | FGF7 -> AP-1/CREB/CREBBP/MYC Expression Targets |
|  | FGF2 -> AP-1/CREB/CREBBP/ELK/SRF/MYC Expression Targets |
|  | FGFR -> AP-1/CREB/CREBBP/ELK/SRF/MYC Signaling |
|  | FrizzledR -> JUN/PAX2 Signaling |
|  | ADCYAP1 Expression Targets |
|  | Glutamate/Gq Expression Targets |
|  | VIP Expression Targets |
|  | HBEGF -> AP-1/ATF Expression Targets |
|  | HBEGF -> TP53 Expression Targets |
|  | HBEGF -> MEF/MYOD Expression Target |
|  | HBEGF -> CREB/MYC Expression Targets |
|  | HGF -> AP-1/CREB/ELK/SRF/MYC Expression Targets |
|  | HGFR -> AP-1/CREB/MYC Signaling |
|  | TAM Receptor Signaling Activation in Cancer |
|  | RAS/RAF/MAPK Signaling Activation in Cancer |
|  | STAT3 and NFkB Activate Inflammation-Induced Tumorigenesis |
|  | RAS/RAF/MAPK Signaling Activation by Blocking of Tumor Suppressors |
|  | TGFB Signaling activation by Blocking of Tumor Suppressors |
|  | VEGFA Dependent Angiogenesis in Cancer |
|  | Inhibition of THBS1 Activates Angiogenesis in Cancer |
|  | VEGF Independent Angiogenesis in Cancer |
|  | CDH2 Activation Promotes Cancer Cell Migration and Survival |
|  | TGFB Family in Epithelial to Mesenchymal Transition in Cancer |
|  | IGF1 -> MEF/MYOD/MYOG Expression Targets |
|  | IGF2/MEF/MYOD Expression Targets |
|  | Insulin -> MEF/MYOD Expression Targets |
|  | AGER -> CREB/SP1 Signaling |
|  | ICAM1 -> AP-1/CREB/ELK/SRF Signaling |
|  | IGF1R -> MEF/MYOD/MYOG Signaling |
|  | FibronectinR -> NF-kB Signaling |
|  | FibronectinR -> AP-1/ELK/SRF/SREBF Signaling |
|  | Fibronectin Expression Targets |
|  | CTGF -> AP-1/CREB/MYC Expression Targets |
|  | CTGF -> NCOR2 Expression Target |
|  | ICAM1 Expression Targets |
|  | IL1R -> STAT3 Signaling |
|  | IL1R -> NF-kB Signaling |
|  | IL16-> ATF/CREB/CREBBP Expression Target |
|  | LPA Expression Targets |
|  | PAF/Gq -> AP-1/ATF1/CREB/ERK/SRF Expression Targets |
|  | Dronabinol/Anandamide Expression Targets |
|  | PAF/Gq -> NF-kB Expression Targets |
|  | S1P Expression Targets |
|  | MTNR1 Signaling |
|  | NRG1 -> MEF/MYOD Expression Targets |
|  | NRG1 -> CREB/CREBBP/ELK/SRF/MYC Expression Targets |
|  | NRG1 -> TP53 Expression Target |
|  | NRG1 -> AP-1/ATF Expression Targets |
|  | NGF -> SMAD3/NF-kB Expression Targets |
|  | NGF -> AP-1/TP53/MYC Expression Targets |
|  | NGF -> FOXO/MYCN/ELK-SRF Expression Targets |
|  | BDNF Expression Targets |
|  | NTF3 Expression Targets |
|  | NTF4 Expression Targets |
|  | NGF -> CREB/CEBPB/MEF2A Expression Targets |
|  | NOTCH -> TCF3 Signaling |
|  | NOTCH Expression Targets |
|  | PDGFB -> AP-1/CREB/MYC Expression Target |
|  | PDGFC -> CREB Expression Target |
|  | PDGFD -> AP-1 Expression Targets |
|  | PDGF -> AP-1/CREB/CREBBP/MYC Expression Targets |
|  | KIT -> MITF Signaling |
|  | PDGFR -> AP-1/MYC Signaling |
|  | AVP/Gs -> CREB/ELK/SRF/AP-1/EGR Expression Targets |
|  | GNRH1 Expression Targets |
|  | GNRH2 Expression Targets |
|  | EDN1 Expression Targets |
|  | AVP/Gs -> MEF/MYOD/NFATC/MYOG Expression Targets |
|  | AVP/Gq -> MEF/MYOD/NFATC/MYOG Expression Targets |
|  | EDN3 Expression Targets |
|  | AVP/Gq -> CREB/ELK/SRF/AP-1/EGR Expression Targets |
|  | PDCD1 -> ATF/CREB/CREBBP Expression Targets |
|  | AngiopoietinR -> AP-1 Signaling |
|  | GDNF -> HSF1 Signaling |
|  | EphrinB -> MAPK/JUN/FOS Signaling |
|  | NTRK -> AP-1/CREB/ELK/SRF/MYC/SMAD3/TP53 Signaling |
|  | GAS6 -> AP-1/CREB Expression Targets |
|  | FIGF -> AP-1 Expression Target |
|  | FIGF -> NCOR2 Expression Target |
|  | KITLG -> AP-1/CREB/CREBBP/MYC Expression Targets |
|  | GDNF -> HSF1 Expression Targets |
|  | CSF1 -> AP-1/CREB/CREBBP/MYC Expression Targets |
|  | PGF -> AP-1/CREB/CREBBP/MYC Expression Targets |
|  | FLT3LG -> AP-1/CREB/CREBBP Expression Targets |
|  | TNF-alpha/TNFRSF1A Signaling |
|  | JNK/MAPK Signaling |
|  | MERTK Signaling |
|  | Ras Signaling |
|  | TGF-beta Signaling |
|  | P38 MAPK/MAPK14 Signaling |
|  | AXL Receptor Tyrosine Kinase Signaling |
|  | PI3K/RAC1 Signaling |
|  | VEGF Signaling |
|  | EGFR Signaling |
|  | WNT Planar Cell Polarity (PCP) non-Canonical Signaling |
|  | ERK/MAPK Canonical Signaling |
|  | TCR -> CREB/CREBBP/ATF Expression Targets |
|  | T-Cell Receptor -> ATF/CREB Signaling |
|  | TGFA -> TP53 Expression Targets |
|  | TGFA -> MEF/MYOD/NFATC Expression Targets |
|  | TGFA/AP-1/ATF Expression Targets |
|  | TGFA -> CREB/CREBBP/ELK-SRF/MYC Expression Targets |
|  | FASLG Expression Targets |
|  | TNFSF10 Expression Targets |
|  | LTA Expression Targets |
|  | TNFSF14 Expression Targets |
|  | TNF -> AP-1 Expression Targets |
|  | CD72 -> AP-1 Expression Targets |
|  | CD40LG -> NF-kB/ELK/SRF -> CREB/NFATC Expression Targets |
|  | TNFSF13 Expression Targets |
|  | TNFSF13B Expression Targets |
|  | TNF -> ELK-SRF Expression Targets |
|  | TNF -> CREB Expression Targets |
|  | TNF -> TP53/ATF Expression Targets |
|  | EDA Expression Targets |
|  | EctodysplasinR -> AP-1 Signaling |
|  | TNFR -> CREB/ELK-SRF Signaling |
|  | TNFR -> AP-1/ATF/TP53 Signaling |
|  | NGFR -> AP-1/CEBPB/CREB/ELK/SRF/TP53 Signaling |
|  | TNFRSF5/6 -> RB1/E2F Signaling |
|  | NGFR -> MEF Signaling |
|  | TNFRSF6 -> DDIT3 Signaling |
|  | TNFRSF6 -> HSF1 Signaling |
|  | TNFRSF1A -> AP-1/ATF/TP53 Signaling |
|  | TNFRSF1A -> CREB/ELK-SRF Signaling |
|  | Thrombopoietin -> SPI1 Expression Targets |
|  | Thrombopoietin -> AP-1/CREB/CREBBP/MYC Expression Targets |
|  | IL1B -> PGE2 Expression Targets |
|  | IL1B Expression Targets |
|  | IL1A Expression Targets |
|  | VEGFA -> ATF/CREB/ELK-SRF Expression Targets |
|  | VEGFA -> NFATC Expression Targets |
|  | VEGFC -> ATF Expression Target |
|  | VEGFA -> AP-1/CREBBP/MYC Expression Targets |
|  | VEGFA -> NCOR2 Expression Target |
|  | VEGFR -> NFATC Signaling |
|  | VEGFR -> AP-1/CREB/MYC Signaling |
|  | VEGFR -> ATF/CREB/ELK-SRF Signaling |
|  | WNT1 Expression Targets |
|  | WNT3A Expression Targets |
|  | WNT4 Expression Targets |
|  | WNT5A Expression Targets |
|  | WNT7A Expression Targets |
|  | ace2 |
|  | PPAR Psoriasis |
|  | Model of PPARG signaling in psoriasis |
|  | PPARG negative regulators and targets |
|  | Model of PPARG related pathways in psoriasis (short version) |
|  | 1\_Inflammation in keratinocytes in the pathology of acne |
|  | 2\_Acute phase initiation in atopic dermatitis |
|  | 1\_Differentiation of psoriatic T cells |
|  | 2\_Interleukin-17 and interleukin-22 signaling in psoriasis |
|  | 1\_TNF and IL1B provoke ECM degradation in osteoarthritis |
|  | 1\_Osteoclast-mediated bone resorption in postmenopausal women |
|  | 1\_Formation of cysts in polycystic kidney disease |
|  | 3\_The role of interstitial fibroblasts in pyelonephritis |
|  | 3\_Enhanced angiogenesis in endometriosis |
|  | 1\_Disruption of luteinizing hormone and follicle-stimulating hormone secretion in PCOS |
|  | 2\_Pathogens stimulate mucins expression in the middle ear |
|  | 1\_Cigarette smoke causes dysfunction and production of inflammatory mediators in alveolar epithelial cells |
|  | 3\_Role of alveolar macrophages in pulmonary tissue destruction in COPD |
|  | 4\_2\_Airway smooth muscle cells proliferation and airway tissue remodeling |
|  | 4\_1\_Polymorphisms associated with inflammatory bowel diseases |
|  | 6\_p12I Viral protein promotes T-cell activation in HTLV-1 infection |
|  | 2\_HIV - induced depletion of CD4+ T-cells |
|  | 1\_Microangiopathy in diabetic neuropathy |
|  | Model of PPARG signaling in psoriais (tested) |
|  | mapk1 protein modificators |
|  | Differentiation of psoriatic T cells |
|  | Interleukin-17 and interleukin-22 signaling in psoriasis |

---

|  |  |
| --- | --- |
| Source | curated |
|  | ResNet |
|  | GO component |

---

|  |  |
| --- | --- |
| MedScan ID | 4214 |

---

|  |  |
| --- | --- |
| LocusLink ID | 4214 |
|  | 26401 |
|  | 116667 |
|  | 100912399 |
|  | 17269 |
|  | 523962 |
|  | 564899 |

---

|  |  |
| --- | --- |
| Alias | SRXY6 |
|  | MAPK/ERK kinase kinase I |
|  | mitogen-activated protein kinase kinase kinase 1, E3 ubiquitin protein ligase |
|  | MEKK |
|  | OTTMUSP00000021865 |
|  | LOC100912399 |
|  | mitogen activated protein kinase kinase kinase 1 |
|  | mitogen-activated protein kinase kinase kinase I |
|  | MAPKKK I |
|  | MEKK1 |
|  | mitogen-activated protein kinase kinase kinase 1-like |
|  | OTTHUMP00000065818 |
|  | Map3k I |
|  | Map3k1 |
|  | MEK kinase 1 |
|  | MAP kinase kinase kinase 1 |
|  | MAPK/ERK kinase kinase 1 |
|  | MAP/ERK kinase kinase 1 |
|  | MAPKKK1 |
|  | MEK kinase I |
|  | MAP/ERK kinase kinase I |
|  | MEK kinase |
|  | MEKK 1 |
|  | mitogen-activated protein kinase kinase kinase 1 |
|  | MAPK/ ERK kinase kinase I |
|  | MAPK/ ERK kinase kinase 1 |
|  | MEKK I |
|  | scaffold |
|  | chromosome |
|  | similar to hCG40615 |
|  | LOC564899 |

---

|  |  |
| --- | --- |
| EC Number | 2.7.11.25 |
|  | 2.7.1.- |

---

|  |  |
| --- | --- |
| GO ID | 0005524 |
|  | 0004709 |
|  | 0004672 |
|  | 0019901 |
|  | 0004674 |
|  | 0008270 |
|  | 0038095 |
|  | 0000165 |
|  | 0002755 |
|  | 0000186 |
|  | 0032147 |
|  | 0071260 |
|  | 0006468 |
|  | 0023014 |
|  | 0031098 |
|  | 0005737 |
|  | 0005829 |
|  | 0008432 |
|  | 0004706 |
|  | 0008092 |
|  | 0016301 |
|  | 0046872 |
|  | 0051019 |
|  | 0031434 |
|  | 0000166 |
|  | 0046625 |
|  | 0016740 |
|  | 0004842 |
|  | 0007254 |
|  | 0007257 |
|  | 0008637 |
|  | 0043010 |
|  | 0003382 |
|  | 0061029 |
|  | 0002011 |
|  | 0016310 |
|  | 0030838 |
|  | 0030334 |
|  | 0007179 |
|  | 0042060 |
|  | 0030054 |
|  | 0005856 |
|  | 0031941 |
|  | 0016020 |
|  | 0007256 |
|  | 0032232 |
|  | 0018105 |
|  | 0043507 |
|  | 0043406 |
|  | 0043065 |
|  | 0045944 |
|  | 0050434 |
|  | 0046777 |
|  | 0051259 |
|  | 0000209 |
|  | 0006970 |
|  | 0006511 |
|  | 0007346 |
|  | 0004702 |
|  | 0006915 |
|  | 0042787 |
|  | 0008545 |
|  | 0030036 |
|  | 0045087 |
|  | 0035556 |
|  | 0034166 |
|  | 0034134 |
|  | 0034142 |
|  | 0034146 |
|  | 0034162 |
|  | 0038123 |
|  | 0038124 |
|  | 0002224 |
|  | 0016772 |
|  | 0016567 |
|  | 0005622 |
|  | 0008063 |
|  | 0034130 |
|  | 0004710 |
|  | 0010552 |
|  | 0005624 |
|  | 0008152 |
|  | 0007165 |
|  | 0042802 |
|  | 0000287 |
|  | 0007249 |
|  | 0005575 |
|  | 0005515 |
|  | 0008372 |
|  | 0001747 |
|  | 0000151 |

---

|  |  |
| --- | --- |
| KEGG ID | hsa:4214 |
|  | rno:116667 |
|  | mmu:26401 |

---

|  |  |
| --- | --- |
| MedLine Reference | 12941696 |
|  | 9808624 |
|  | 11784851 |
|  | 12011049 |
|  | 12049732 |
|  | 15037605 |
|  | 15164763 |
|  | 16006144 |
|  | 16427076 |
|  | 16491099 |
|  | 8643568 |
|  | 10349636 |
|  | 10465784 |
|  | 10523642 |
|  | 10805784 |
|  | 10852963 |
|  | 11042159 |
|  | 11076861 |
|  | 11217851 |
|  | 11756439 |
|  | 11782455 |
|  | 11891332 |
|  | 12048245 |
|  | 12122119 |
|  | 12458213 |
|  | 12466851 |
|  | 12493778 |
|  | 12584189 |
|  | 12839996 |
|  | 12878610 |
|  | 14500727 |
|  | 14586403 |
|  | 14967141 |
|  | 15001576 |
|  | 15358865 |
|  | 15558021 |
|  | 15601830 |
|  | 15607429 |
|  | 15795331 |
|  | 15882989 |
|  | 16141072 |
|  | 16141073 |
|  | 16557301 |
|  | 16568086 |
|  | 16760432 |
|  | 16761309 |
|  | 16887003 |
|  | 17101801 |
|  | 17143273 |
|  | 4035594 |
|  | 4724491 |
|  | 4865553 |
|  | 5630254 |
|  | 6740513 |
|  | 6883178 |
|  | 7618106 |
|  | 8385802 |
|  | 8597633 |
|  | 8621675 |
|  | 8637721 |
|  | 9427625 |
|  | 9924790 |
|  | 10611349 |
|  | 11746500 |
|  | 11815602 |
|  | 11903060 |
|  | 11971970 |
|  | 11971971 |
|  | 12079429 |
|  | 12185592 |
|  | 12228228 |
|  | 12456688 |
|  | 12600818 |
|  | 12763138 |
|  | 14612408 |
|  | 14743216 |
|  | 15205333 |
|  | 15299005 |
|  | 16044153 |
|  | 16046415 |
|  | 16286467 |
|  | 16434970 |
|  | 16636664 |
|  | 16714289 |
|  | 9008162 |
|  | 9689078 |
|  | 9836645 |
|  | 16344560 |
|  | 17301822 |
|  | 17397260 |
|  | 16081685 |
|  | 9078260 |

---

|  |  |
| --- | --- |
| Organism | Homo sapiens {Organism urn:agi-taxid:9606} |
|  | Mus musculus {Organism urn:agi-taxid:10090} |
|  | Rattus norvegicus {Organism urn:agi-taxid:10116} |
|  | Homo sapiens |
|  | Mus musculus |
|  | Rattus norvegicus |
|  | Bos taurus |
|  | Danio rerio |
|  | Human |

---

|  |  |
| --- | --- |
| Mouse chromosome position | 13 63.36 cM |
|  | 13 60.0 cM |

---

|  |  |
| --- | --- |
| OMIM ID | 600982 |
|  | 613762 |
|  | OMIM:600982 |

---

|  |  |
| --- | --- |
| Rat chromosome position | 2q14 |
|  | 2 |

---

|  |  |
| --- | --- |
| Hugo ID | 6848 |
|  | HGNC:6848 |

---

|  |  |
| --- | --- |
| Human chromosome position | 5q11.2 |

---

|  |  |
| --- | --- |
| Swiss-Prot Accession | Q13233 |
|  | Q13233.4 |
|  | F8VQ72 |
|  | P53349.3 |
|  | Q62925 |
|  | Q62925.1 |
|  | F1M778 |
|  | P53349 |
|  | M3K1\_HUMAN |
|  | M3K1\_RAT |

---

|  |  |
| --- | --- |
| PIR ID | G01887 |
|  | T10757 |

---

|  |  |
| --- | --- |
| GenBank ID | NC\_000005 |
|  | XR\_001742068 |
|  | NM\_005921 |
|  | NP\_005912 |
|  | XM\_017009485 |
|  | XP\_016864974 |
|  | XM\_017009484 |
|  | XP\_016864973 |
|  | NG\_031884 |
|  | AC008937 |
|  | HF583845 |
|  | CCQ43342 |
|  | U29671 |
|  | AAB05828 |
|  | AA602425 |
|  | AF042838 |
|  | AAC97073 |
|  | AK307828 |
|  | BU194120 |
|  | BX537527 |
|  | DA889202 |
|  | Q13233 |
|  | NC\_000079 |
|  | NM\_011945 |
|  | NP\_036075 |
|  | XM\_017315516 |
|  | XP\_017171005 |
|  | XM\_017315517 |
|  | XP\_017171006 |
|  | AC118704 |
|  | CH466568 |
|  | EDL18419 |
|  | EDL18420 |
|  | U29669 |
|  | U29670 |
|  | AB014614 |
|  | BAA85878 |
|  | AF117340 |
|  | AAD25049 |
|  | AK142245 |
|  | L13103 |
|  | AAA97500 |
|  | U23470 |
|  | AAA85038 |
|  | P53349 |
|  | NC\_005101 |
|  | NM\_053887 |
|  | NP\_446339 |
|  | AC111653 |
|  | CH473955 |
|  | EDM10340 |
|  | EDM10341 |
|  | EDM10342 |
|  | JQ013733 |
|  | AFD32168 |
|  | U48596 |
|  | AAC52596 |
|  | Q62925 |
|  | XM\_006231926 |
|  | XP\_006231988 |
|  | AC\_000070 |
|  | XM\_006224013 |
|  | XP\_006224075 |
|  | AABR07008108 |
|  | AAHX01013021 |
|  | AAHX01013022 |
|  | XM\_011543407 |
|  | XP\_011541709 |
|  | XM\_011543408 |
|  | XP\_011541710 |
|  | XM\_011543406 |
|  | XP\_011541708 |
|  | XM\_005248519 |
|  | XP\_005248576 |
|  | NC\_018916 |
|  | AMYH02011332 |
|  | XM\_006517664 |
|  | XP\_006517727 |
|  | XM\_011244663 |
|  | XP\_011242965 |
|  | XM\_011244664 |
|  | XP\_011242966 |
|  | AC\_000035 |
|  | AAHY01110126 |
|  | AAHY01110127 |
|  | AAHY01110128 |
|  | AAHY01110129 |
|  | AAHY01110130 |
|  | BC172631 |
|  | AAI72631 |
|  | XM\_005248520 |
|  | XP\_005248577 |
|  | AC\_000137 |
|  | ABBA01015768 |
|  | XM\_006517665 |
|  | XP\_006517728 |
|  | XM\_006517667 |
|  | XP\_006517730 |
|  | XM\_006517666 |
|  | XP\_006517729 |
|  | XM\_006231919 |
|  | XP\_006231981 |
|  | XM\_006231920 |
|  | XP\_006231982 |
|  | AABR06012815 |
|  | NT\_006713 |
|  | NW\_922607 |
|  | NW\_001838934 |
|  | AC\_000048 |
|  | NT\_039590 |
|  | NW\_001030537 |
|  | NW\_001084795 |
|  | NW\_047620 |
|  | XM\_001074670 |
|  | XP\_001074670 |
|  | XP\_042066 |
|  | AI317205 |
|  | XM\_042066 |
|  | 34854322 |
|  | 16758759 |
|  | 16758760 |
|  | 1354136 |
|  | 1354137 |
|  | 2499638 |
|  | 51767945 |
|  | 33859655 |
|  | 33859656 |
|  | 6141558 |
|  | 6141559 |
|  | 4583379 |
|  | 4583380 |
|  | 4032472 |
|  | 293729 |
|  | 293730 |
|  | 1143819 |
|  | 1143820 |
|  | 19860665 |
|  | ENST00000264777 |
|  | 51465008 |
|  | 51464911 |
|  | 51464912 |
|  | 1002603 |
|  | 1002604 |
|  | 2815887 |
|  | 2815888 |
|  | 31873589 |
|  | 8488988 |
|  | XM\_001128827 |
|  | XP\_001128827 |
|  | NC\_007318 |
|  | NW\_001493936 |
|  | XM\_602275 |
|  | XP\_602275 |
|  | NC\_007123 |
|  | NW\_001510849 |
|  | XM\_688224 |
|  | XP\_693316 |
|  | 148887396 |
|  | 148686472 |
|  | 148686473 |
|  | 149059333 |
|  | 149059334 |
|  | 149059335 |

---

|  |  |
| --- | --- |
| Swiss-Prot ID | M3K1\_HUMAN |
|  | M3K1\_RAT |

---

|  |  |
| --- | --- |
| Cell Localization | Membrane |
|  | Cytoplasm |

---

|  |  |
| --- | --- |
| Ensembl ID | ENSG00000095015 |
|  | ENSP00000382423.3 |
|  | ENST00000399503.4 |
|  | ENSMUSG00000021754 |
|  | ENSMUSP00000104890.2 |
|  | ENSMUST00000109267.8 |
|  | ENSRNOG00000013177 |
|  | ENSRNOP00000017968.3 |
|  | ENSRNOT00000017968.3 |
|  | ENSRNOG00000050275 |
|  | ENSRNOP00000063470.3 |
|  | ENSRNOT00000065473.4 |
|  | ENST00000399503.3 |
|  | ENSP00000382423 |
|  | ENST00000399503 |
|  | ENSMUSP00000104890 |
|  | ENSMUST00000109267 |
|  | ENSRNOP00000017968 |
|  | ENSRNOT00000017968 |
|  | ENSRNOP00000063470 |
|  | ENSRNOT00000065473 |

---

|  |  |
| --- | --- |
| MGI ID | MGI:1346872 |
|  | 1346872 |
|  | 104845 |

---

|  |  |
| --- | --- |
| RGD ID | 620966 |
|  | 6487511 |

---

|  |  |
| --- | --- |
| Unigene ID | Rn.11081 |
|  | Mm.15918 |
|  | Hs.653654 |
|  | Hs.657756 |
|  | Hs.508461 |
|  | Hs.170610 |
|  | Hs.634810 |
|  | Bt.65484 |
|  | Dr.87896 |
|  | Mm.446367 |

---

|  |  |
| --- | --- |
| Homologene ID | 8056 |

---

|  |  |
| --- | --- |
| Shape | Sickle-vertex |

---

|  |  |
| --- | --- |
| IPI ID | IPI00210195 |
|  | IPI00855985 |
|  | IPI00130375 |
|  | IPI00677379 |

---

|  |  |
| --- | --- |
| Chromosome position | 2q14 |
|  | 13 60.0 cM |
|  | 5q11.2 |

---

|  |  |
| --- | --- |
| Microarray ID | 1368871\_at |
|  | u48596\_at |
|  | u48596\_g\_at |
|  | A\_43\_P12971 |
|  | A\_51\_P402193 |
|  | A\_65\_P05116 |
|  | AF042838 >1 |
|  | AF042838 >2 |
|  | 33009\_at |
|  | 214786\_at |
|  | 225927\_at |
|  | 2004\_at |
|  | A\_23\_P41796 |
|  | A\_24\_P766204 |
|  | OR2370 |
|  | OH5738A |
|  | ST079 |
|  | MA256 |
|  | RA0812 |
|  | 103020\_s\_at |
|  | 103021\_r\_at |
|  | 103022\_at |
|  | 243030\_at |

---

|  |  |
| --- | --- |
| Homo sapiens Chromosome position | 5q11.2 |

---

|  |  |
| --- | --- |
| Ensembl Transcript ID | ENST00000399503 |

---

|  |  |
| --- | --- |
| ENSEMBL | ENSG00000095015 |

---

|  |  |
| --- | --- |
| FunctionalClass | ATP binding |
|  | MAP kinase kinase kinase |
|  | identical protein binding |
|  | protein binding |
|  | kinase |
|  | magnesium ion binding |
|  | metal ion binding |
|  | nucleotide binding |
|  | protein kinase |
|  | protein serine/threonine kinase |
|  | transferase |
|  | zinc ion binding |
|  | ubiquitin-protein ligase |
|  | JUN kinase kinase |

---

|  |  |
| --- | --- |
| Hugo Symbol | MAP3K1 |

---

|  |  |
| --- | --- |
| Bos taurus Chromosome position | 20 |

---

|  |  |
| --- | --- |
| Mus musculus Chromosome position | 13 60.0 cM |

---

|  |  |
| --- | --- |
| KEGG pathway | GnRH signaling pathway |
|  | MAPK signaling pathway |

---

|  |  |
| --- | --- |
| Rattus norvegicus Chromosome position | 2q14 |

---

|  |  |
| --- | --- |
| Ensembl Gene ID | ENSG00000095015 |

---

|  |  |
| --- | --- |
| ProteinAtlas NormalTissue | Tissue: lymph node; Cell type: non-germinal center cells; Level: Moderate; Expression type: Staining; Reliability: Supportive |
|  | Tissue: bone marrow; Cell type: hematopoietic cells; Level: Moderate; Expression type: Staining; Reliability: Supportive |
|  | Tissue: bronchus; Cell type: respiratory epithelial cells; Level: Strong; Expression type: Staining; Reliability: Supportive |
|  | Tissue: uterus |
|  | post-menopause; Cell type: cells in endometrial stroma; Level: Moderate; Expression type: Staining; Reliability: Supportive |
|  | Tissue: parathyroid gland; Cell type: glandular cells; Level: Strong; Expression type: Staining; Reliability: Supportive |
|  | Tissue: lateral ventricle; Cell type: glial cells; Level: Moderate; Expression type: Staining; Reliability: Supportive |
|  | Tissue: appendix; Cell type: lymphoid tissue; Level: Moderate; Expression type: Staining; Reliability: Supportive |
|  | Tissue: colon; Cell type: glandular cells; Level: Strong; Expression type: Staining; Reliability: Supportive |
|  | Tissue: vagina; Cell type: squamous epithelial cells; Level: Moderate; Expression type: Staining; Reliability: Supportive |
|  | Tissue: pancreas; Cell type: islets of Langerhans; Level: Moderate; Expression type: Staining; Reliability: Supportive |
|  | Tissue: smooth muscle; Cell type: smooth muscle cells; Level: Weak; Expression type: Staining; Reliability: Supportive |
|  | Tissue: adrenal gland; Cell type: glandular cells; Level: Strong; Expression type: Staining; Reliability: Supportive |
|  | Tissue: appendix; Cell type: glandular cells; Level: Moderate; Expression type: Staining; Reliability: Supportive |
|  | Tissue: breast; Cell type: glandular cells; Level: Moderate; Expression type: Staining; Reliability: Supportive |
|  | Tissue: cerebellum; Cell type: cells in granular layer; Level: Moderate; Expression type: Staining; Reliability: Supportive |
|  | Tissue: cerebellum; Cell type: cells in molecular layer; Level: Moderate; Expression type: Staining; Reliability: Supportive |
|  | Tissue: cerebellum; Cell type: Purkinje cells; Level: Moderate; Expression type: Staining; Reliability: Supportive |
|  | Tissue: cerebral cortex; Cell type: glial cells; Level: Weak; Expression type: Staining; Reliability: Supportive |
|  | Tissue: cerebral cortex; Cell type: neuronal cells; Level: Moderate; Expression type: Staining; Reliability: Supportive |
|  | Tissue: cervix, uterine; Cell type: glandular cells; Level: Strong; Expression type: Staining; Reliability: Supportive |
|  | Tissue: cervix, uterine; Cell type: squamous epithelial cells; Level: Moderate; Expression type: Staining; Reliability: Supportive |
|  | Tissue: duodenum; Cell type: glandular cells; Level: Strong; Expression type: Staining; Reliability: Supportive |
|  | Tissue: epididymis; Cell type: glandular cells; Level: Moderate; Expression type: Staining; Reliability: Supportive |
|  | Tissue: esophagus; Cell type: squamous epithelial cells; Level: Moderate; Expression type: Staining; Reliability: Supportive |
|  | Tissue: fallopian tube; Cell type: glandular cells; Level: Moderate; Expression type: Staining; Reliability: Supportive |
|  | Tissue: gall bladder; Cell type: glandular cells; Level: Moderate; Expression type: Staining; Reliability: Supportive |
|  | Tissue: heart muscle; Cell type: myocytes; Level: Strong; Expression type: Staining; Reliability: Supportive |
|  | Tissue: hippocampus; Cell type: glial cells; Level: Weak; Expression type: Staining; Reliability: Supportive |
|  | Tissue: hippocampus; Cell type: neuronal cells; Level: Moderate; Expression type: Staining; Reliability: Supportive |
|  | Tissue: kidney; Cell type: cells in glomeruli; Level: Negative; Expression type: Staining; Reliability: Supportive |
|  | Tissue: kidney; Cell type: cells in tubules; Level: Moderate; Expression type: Staining; Reliability: Supportive |
|  | Tissue: liver; Cell type: bile duct cells; Level: Weak; Expression type: Staining; Reliability: Supportive |
|  | Tissue: liver; Cell type: hepatocytes; Level: Strong; Expression type: Staining; Reliability: Supportive |
|  | Tissue: lung; Cell type: macrophages; Level: Strong; Expression type: Staining; Reliability: Supportive |
|  | Tissue: lung; Cell type: pneumocytes; Level: Weak; Expression type: Staining; Reliability: Supportive |
|  | Tissue: lymph node; Cell type: germinal center cells; Level: Moderate; Expression type: Staining; Reliability: Supportive |
|  | Tissue: nasopharynx; Cell type: respiratory epithelial cells; Level: Strong; Expression type: Staining; Reliability: Supportive |
|  | Tissue: oral mucosa; Cell type: squamous epithelial cells; Level: Weak; Expression type: Staining; Reliability: Supportive |
|  | Tissue: ovary; Cell type: follicle cells; Level: Weak; Expression type: Staining; Reliability: Supportive |
|  | Tissue: ovary; Cell type: ovarian stroma cells; Level: Weak; Expression type: Staining; Reliability: Supportive |
|  | Tissue: pancreas; Cell type: exocrine glandular cells; Level: Strong; Expression type: Staining; Reliability: Supportive |
|  | Tissue: placenta; Cell type: decidual cells; Level: Strong; Expression type: Staining; Reliability: Supportive |
|  | Tissue: placenta; Cell type: trophoblastic cells; Level: Moderate; Expression type: Staining; Reliability: Supportive |
|  | Tissue: prostate; Cell type: glandular cells; Level: Moderate; Expression type: Staining; Reliability: Supportive |
|  | Tissue: rectum; Cell type: glandular cells; Level: Moderate; Expression type: Staining; Reliability: Supportive |
|  | Tissue: salivary gland; Cell type: glandular cells; Level: Moderate; Expression type: Staining; Reliability: Supportive |
|  | Tissue: seminal vesicle; Cell type: glandular cells; Level: Moderate; Expression type: Staining; Reliability: Supportive |
|  | Tissue: skeletal muscle; Cell type: myocytes; Level: Weak; Expression type: Staining; Reliability: Supportive |
|  | Tissue: skin; Cell type: epidermal cells; Level: Moderate; Expression type: Staining; Reliability: Supportive |
|  | Tissue: small intestine; Cell type: glandular cells; Level: Moderate; Expression type: Staining; Reliability: Supportive |
|  | Tissue: spleen; Cell type: cells in red pulp; Level: Moderate; Expression type: Staining; Reliability: Supportive |
|  | Tissue: spleen; Cell type: cells in white pulp; Level: Moderate; Expression type: Staining; Reliability: Supportive |
|  | Tissue: stomach, lower; Cell type: glandular cells; Level: Moderate; Expression type: Staining; Reliability: Supportive |
|  | Tissue: stomach, upper; Cell type: glandular cells; Level: Moderate; Expression type: Staining; Reliability: Supportive |
|  | Tissue: testis; Cell type: cells in seminiferus ducts; Level: Moderate; Expression type: Staining; Reliability: Supportive |
|  | Tissue: testis; Cell type: Leydig cells; Level: Moderate; Expression type: Staining; Reliability: Supportive |
|  | Tissue: thyroid gland; Cell type: glandular cells; Level: Strong; Expression type: Staining; Reliability: Supportive |
|  | Tissue: tonsil; Cell type: germinal center cells; Level: Moderate; Expression type: Staining; Reliability: Supportive |
|  | Tissue: tonsil; Cell type: non-germinal center cells; Level: Strong; Expression type: Staining; Reliability: Supportive |
|  | Tissue: tonsil; Cell type: squamous epithelial cells; Level: Moderate; Expression type: Staining; Reliability: Supportive |
|  | Tissue: urinary bladder; Cell type: urothelial cells; Level: Moderate; Expression type: Staining; Reliability: Supportive |
|  | Tissue: uterus, post-menopause; Cell type: cells in endometrial stroma; Level: Moderate; Expression type: Staining; Reliability: Supportive |
|  | Tissue: uterus, post-menopause; Cell type: glandular cells; Level: Moderate; Expression type: Staining; Reliability: Supportive |
|  | Tissue: uterus, pre-menopause; Cell type: cells in endometrial stroma; Level: Moderate; Expression type: Staining; Reliability: Supportive |
|  | Tissue: uterus, pre-menopause; Cell type: glandular cells; Level: Moderate; Expression type: Staining; Reliability: Supportive |
|  | Tissue: vulva/anal skin; Cell type: epidermal cells; Level: Moderate; Expression type: Staining; Reliability: Supportive |

---
